# Neurological features of epilepsy, ataxia, sensorineural deafness, tubulopathy syndrome

**DOI:** 10.1111/dmcn.12171

**Published:** 2013-03-14

**Authors:** J Helen Cross, Ruchi Arora, Rolf A Heckemann, Roxana Gunny, Kling Chong, Lucinda Carr, Torsten Baldeweg, Ann-Marie Differ, Nicholas Lench, Sophie Varadkar, Tony Sirimanna, Evangeline Wassmer, Sally A Hulton, Milos Ognjanovic, Venkateswaran Ramesh, Sally Feather, Robert Kleta, Alexander Hammers, Detlef Bockenhauer

**Affiliations:** 1 Neurosciences Unit, Great Ormond Street Hospital for Children NHS Trust and UCL-Institute of Child HealthLondon, UK; 2 The Neurodis FoundationLyon, France; 3 Department of Radiology, Great Ormond Street Hospital for Children NHS Trust and UCL-Institute of Child HealthLondon, UK; 4 Developmental Cognitive Neuroscience Unit, Great Ormond Street Hospital for Children NHS Trust and UCL-Institute of Child HealthLondon, UK; 5 Department of Molecular Genetics, Great Ormond Street Hospital for Children NHS Trust and UCL-Institute of Child HealthLondon, UK; 6 Department of Audiology, Great Ormond Street Hospital for Children NHS Trust and UCL-Institute of Child HealthLondon, UK; 7 Birmingham Children's HospitalBirmingham, UK; 8 Great North Children's HospitalNewcastle upon Tyne, UK; 9 Leeds Teaching Hospitals/University of LeedsLeeds, UK; 10 Department of Nephrology, Great Ormond Street Hospital for Children NHS Trust and UCL-Institute of Child HealthLondon, UK; 11 Division of Experimental Medicine, Imperial College LondonLondon, UK

## Abstract

**AIM:**

Recently, we reported a previously unrecognized symptom constellation comprising epilepsy, ataxia, sensorineural deafness, and tubulopathy (EAST syndrome) associated with recessive mutations in the *KCNJ10* gene. Here, we provide a detailed characterization of the clinical features of the syndrome to aid patient management with respect to diagnosis, prognostic counselling, and identification of best treatment modalities.

**METHOD:**

We conducted a retrospective review of the detailed neurological and neuroradiological features of nine children (four females, five males; age range at last examination 6–20y) with genetically proven EAST syndrome.

**RESULTS:**

All children presented with tonic–clonic seizures in infancy. Later, non-progressive, cerebellar ataxia and hearing loss were noted. Whilst seizures mostly responded well to treatment, ataxia proved to be the most debilitating feature, with three patients non-ambulant. All available magnetic resonance imaging (MRI) revealed subtle symmetrical signal changes in the cerebellar dentate nuclei. Moreover, four patients had a small corpus callosum and brainstem hypoplasia, and three had a small spinal cord. Regional quantitative volumetric analysis of the images confirmed the corpus callosum and brainstem hypoplasia and showed further patterns of variation from the norm.

**INTERPRETATION:**

The neurological features of EAST syndrome appear to be non-progressive, which is important for prognostic counselling. The spectrum of EAST syndrome includes consistent abnormalities on brain MRI, which may aid diagnosis. Further longitudinal documentation is required to determine the true natural history of the disorder.

Mutations in genes coding for potassium channels have been associated with a variety of hereditary diseases, including episodic ataxia, deafness, epilepsy, and cardiac arrhythmias.^1^–^5^ *KCNJ10* (also called Kir4.1) encodes an inwardly rectifying potassium channel that is expressed in renal epithelial cells, inner ear cells, and glial cells in the central nervous system.^6^–^8^ Recessive mutations in this gene cause a newly recognized syndrome characterized by epilepsy, ataxia, sensorineural deafness, and tubulopathy (EAST syndrome).^9^,^10^ A similar phenotype, but with neonatal lethality, is seen in KCNJ10 knockout mice.^11^–^13^ Few clinical reports characterizing the phenotype currently exist in the literature; in particular, the nature of the motor disorder and of the epilepsy have not been demonstrated. Here we describe the neurological features of nine children from six families with proven recessive mutations in *KCNJ10*, in whom detailed neurological assessment was performed, including imaging studies. Data were collected under an NHS ethics approved protocol and parents gave informed consent to publication of the report.

## Method

### Participants

The children were referred to Great Ormond Street Hospital for clinical assessment at variable points in their natural history. All patients exhibited the four cardinal symptoms of epilepsy, ataxia, sensorineural deafness, and tubulopathy (Table I).

**Table I:** Summary of clinical features

Patient	Age (y: m)	Sex	First seizure	Current seizure frequency	Anticonvulsants	Seizure recurrence	Hearing loss	Mutations in *KCNJ10*	MRI changes
1-1	16:3	F	TCS at 5mo	Sleep events, probably frontal lobe seizures	Carbamazepine	Sleep events after wean	Yes, hearing aids from 5y	P.R65P/P.R65P	Subtle corpus callosum changes, subtle pontine hypoplasia, signal abnormality in the dentate nuclei, cerebellar hypoplasia
1-2	10:9	M	TCS at 4mo on awakening	Focal seizures	Sodium valproate and lamotrigine	On weaning valproate	Not assessed	P.R65P/P.R65P	Reported as normal
1-3	7:7	F	TCS at 4mo on awakening	Seizure free	Sodium valproate	No	Yes, mild to moderate	P.R65P/P.R65P	None
1-4	6:3	F	GTCS at 4mo on awakening	Seizure free	Sodium valproate	No	Yes, mild to moderate	P.R65P/P.R65P	None
2	16:0	M	GTCS, status epilepticus at 4mo	Focal seizures	Sodium valproate started at 4mo, weaned at 4y	Recurrence at 12y; phenytoin and valproate	Yes, hearing aids from 15y	P.R65P/P.R65P	Pontine hypoplasia, signal abnormality in the dentate nuclei, cerebellar hypoplasia
3	16:7	M	GTCS at 2mo	TCS when unwell	Phenobarbitone; changed to lamotrigine at 7y	No	Yes, hearing aids from 5y	R297C/R297C	Pontine hypoplasia, signal abnormality in the dentate nuclei, cerebellar hypoplasia
4	20:3	M	GTCS at 6mo	TCS when unwell	Carbamazepine, then lamotrigine	Continued	Yes, hearing aids from 2y	P.R65P/*p*.R199X	Non-specific lack of white matter bulk, with pontine hypoplasia. Bilateral symmetrical signal abnormality within the cerebellar hemispheres, white matter and deep cerebellar nuclei
5-1	19:1	F	GTCS at 3mo	Seizure free	Lamotrigine	Seizure free 4–8y. Seizure deterioration at 8y, on weaning valproate	Yes, mild to moderate	P.F75L/P.F75L	Signal abnormality in the dentate nuclei, small focus of non-specific signal abnormality in left thalamus, cerebellar hypoplasia
5-2	10:6	M	GTCS at 6mo	Seizure free	Lamotrigine	Three GTCS at 9mo; focal seizures between 5y and 6y	Yes, hearing aids from 5y	P.F75L/P.F75L	Subtle signal abnormality in both cerebellar dentate nuclei, cerebellar hypoplasia

TCS, tonic–clonic seizure; GTCS, generalized tonic–clonic seizure; MRI, magnetic resonance imaging.

**What this paper adds**

- Detailed delineation of the neurological features including response to AEDs in a large patient cohort.
- Identification of subtle but characteristic MRI changes that may serve as diagnostic criteria.
- Systematic differences in MRI between patients and comparison children identified using a novel approach of volumetric analysis.

Magnetic resonance imaging (MRI) was performed using a 1.5T Siemens Avanto or Symphony scanner (Siemens Healthcare, Camberley, UK) and brain sequences obtained included axial and coronal T2-weighted turbo spin echo (TSE) images (retention time [TR] 4920ms, echo time [TE] 101ms, slice thickness 4mm, matrix 384×245), volumetric three-dimensional (3D) T1-weighted magnetization-prepared with rapid acquisition with gradient echo (MP-RAGE) images acquired sagitally and reconstructed in the orthogonal coronal and axial planes (TR/TE 11/4.9, voxel size 1mm), and volumetric 3D fluid-attenuated inversion recovery (FLAIR) images reconstructed in the axial and coronal planes (TR/TE 6000/318, voxel size 1mm). In the five patients who underwent targeted spine MRI, sagittal T2-weighted TSE (TR/TE 3000/111, slice thickness 3mm) and T1-weighted TSE (TR/TE 465/11, slice thickness 3mm) images were obtained. Further data were obtained from medical records (including those of original presentation); all available electroencephalograms and MRI were reviewed. Mutation analysis and the electrophysiological consequences of identified mutations in these patients have been reported previously,^8^,^9^,^14^,^15^ as have the ophthalmological features.^16^

### Volumetric analysis of MRI

T1-weighted magnetic resonance brain images were available for volumetric analysis for four patients and eight age- and sex-matched comparison children imaged on the same scanner. Volumetric brain image analysis on 83 regions was carried out on the basis of anatomical segmentation using multi-atlas propagation with enhanced registration (MAPER; http://www.soundray.org/maper.)^17^ This automated procedure consists of propagating a number of given atlas label sets onto the target images using non-rigid image registration, with subsequent consolidation of the resulting segmentations into a single label set. The propagation source comprised 30 atlases that had been generated through manual delineation of 83 regions on T1-weighted brain MRI acquired from young, healthy adults. Manual segmentation protocols have been published previously.^18^,^19^ The accuracy and robustness of MAPER as well as the validity of the resulting segmentations have been shown extensively on a variety of normal and abnormal target images.^17^,^20^ An example of region identification by automatic segmentation is given in supplemental figure 1. Normative volumetric reference data were generated by segmenting images from healthy individuals aged between 20 years and 30 years (*n*=83) from the IXI database (http://www.brain-development.org). Labels of cortical regions containing grey matter or white matter portions (i.e. all regions except ventricles, central structures, cerebellum, and brainstem) were multiplied with a binary grey-matter mask. Regional volumes were normalized by intracranial volume. Images of patients and age- and sex-matched comparison children in this study were processed in an identical fashion. Individual region sizes are expressed as score values after z-transformation using the distribution of volumes in the reference database.

## Results

Nine participants (five males, four females) were assessed at ages 6 years 2 months to 20 years of age; five out of the nine probands were offspring of consanguineous families of Pakistani origin. Of the remaining four, two were siblings of Afro-Caribbean origin, one Iranian and one adopted (ethnicity unknown). Detailed individual case histories are below. A summary of the participants' clinical features, as well as their *KCNJ10* mutation status is summarized in Table I. A video demonstrating the movement disorder in one patient (5-2) is provided in the online supplementary material.

### Individual case histories

#### Patient 1-1

The family of patient 1-1 is of Pakistani heritage. This female was born at term after an unremarkable pregnancy by spontaneous vaginal delivery with a birthweight of 3.4kg. She fed and grew well for the first month of life, but then developed irritability, poor feeding, constipation, and failure to thrive, leading to elective admission at 4 months of age. Investigations at that time identified hypokalaemic metabolic alkalosis. Potassium supplementation (8mmol/kg/d) accompanied by indometacin (2mg/kg/d) administration, as well as nasogastric tube feeding, resulted in good weight gain. Hypomagnesaemia with renal magnesium wasting was first noted at 3 years of age.

The first so-called short generalized tonic–clonic seizure (GTCS) was noted at 4 months of age, during the child's first admission to hospital. She experienced multiple GTCS (as recorded) during this hospital stay, which were treated with diazepam, phenytoin, and eventually carbamazepine, which achieved good subsequent seizure control. Surface electroencephalogram (EEG) at 5 months of age was reported as showing spikes and sharp waves localizing to the vertex. She was weaned from carbamazepine at 7 years of age with no seizure recurrence. However, nocturnal events subsequently occurred, and it remains unclear whether these were epileptic in origin. Video-EEG telemetry at 16 years documented three stereotypical events with no preceding EEG changes. The working diagnosis is frontal lobe seizures.

Developmental delay was apparent from the end of her first year: she sat unsupported at 1 year and walked independently at 2 years of age. Moreover, ataxia became apparent with a broad-based gait and intention tremor. At 9 years of age a trial of acetazolamide was given in an attempt to improve the ataxia, but she developed severe headaches and this medication was discontinued. She attends a school for pupils with special needs.

At 5 years of age, she was noted to have hearing problems and was subsequently diagnosed with a sensorineural hearing impairment for which she has been fitted with hearing aids. To date, yearly audiograms show no definite change in the hearing impairment.

At 16 years this patient reveals an ataxic wide-based gait with no apparent truncal ataxia. There is evidence of past-pointing, indicating cerebellar dysfunction. She has bilateral brisk lower limb reflexes with extensor plantar responses and mild facial dystonia, but normal tone elsewhere.

#### Patient 1-2 (distant cousin of patient 1-1 and sibling of patient 1-3 and patient 1-4)

Patient 1-2 was born at term after an uncomplicated pregnancy with a birthweight of 3.5kg. He first presented at 4 months with an apparent GTCS on awakening, lasting 3 to 4 minutes. Treatment with phenytoin was started at 4 months of age, but he continued to experience GTCS. His antiepileptic medication was changed to valproate at 10 months of age, as he was believed to have developed ‘worsening tremors’ from phenytoin. However, his seizures persisted, lasting 3 to 4 minutes and occurring, on average, twice a month. He spontaneously became seizure free between the ages of 4 years and 7 years 6 months. He was weaned from valproate at 7 years 6 months of age and seizures recurred, but were now of a focal nature, involving staring episodes lasting up to 1.5 minutes, occurring two or three times per day, with skin colour change. There have been no GTCS since 4 years of age, but his focal seizures persist. At his last examination, at 10 years of age, he was being treated with lamotrigine and valproate, with the frequency of his focal seizures being unchanged.

Mild hypomagnesaemia at 0.63mmol/L was noted at initial presentation, at which time he showed failure to thrive. Hypokalaemia was first noted at 11 months of age, but attributed to a concurrent gastrointestinal illness. At 3 years of age he presented again with an intercurrent illness; hypokalaemia (2.2mmol/L) and hypomagnesaemia (0.54mmol/L) led to suspicion of a tubulopathy and oral electrolyte supplementation was instituted.

Neurodevelopment had been delayed from the outset: he first sat unsupported at 2 years of age and walked with support at 4 years of age. He has never gained full independent walking. There has been no deterioration of skills acquired, and there is a very slow but progressive improvement in attempting to walk unaided. He was noted to have a coarse tremor from an early age that becomes more apparent when he performs any motor task. He spoke his first meaningful words at 2 years of age, making two-word sentences at 4 years of age. He has slurring of speech with an explosive tone. He attends a mainstream school with support but is currently not achieving. He has moderate learning difficulties, with evolving behavioural difficulties.

He was not recognized to have a hearing impairment. At the age of 9 years, a formal audiological assessment was attempted, but was unsuccessful owing to impacted cerumen and his motoric and behavioural difficulties.

At 9 years of age, this patient exhibits cerebellar signs, with broad-based gait, intention tremor, titubation, dysdiadochokinesia, and dysmetria. He has no nystagmus. His reflexes are normal, with normal tone and no ankle clonus, and flexor plantar response. He has normal sensation, position, and vibration sense, with no skeletal abnormalities.

#### Patient 1-3

This female was born at term after an uncomplicated pregnancy with a birthweight of 3.1kg. She first presented at 4 months of age with GTCS occurring in the early hours of the morning. Biochemical testing at that time revealed mild hypomagnesaemia (0.62mmol/L), but other parameters were normal. Treatment with valproate was commenced immediately because of previous experience with the older sibling (patient 1-2), with good seizure control. She has been seizure free from 4 months of age and she continues to be treated with valproate. The parents noted a coarse tremor from an early age with unsteadiness while attempting any activity. She started walking with hands held at 4 years and has recently started taking a few steps unaided. She has difficulties with fine motor control as a result of tremor, and often uses one hand to support the other while eating or drawing. She spoke her first meaningful words at 1 year 6 months of age and has been making full sentences since 6 years of age. Her speech is slurred with some scanning dysarthria. She attends a mainstream school, where she has demonstrated mild learning difficulties but no behavioural problems.

She was not recognized to have a hearing problem, but audiological testing at the age of 6 years revealed high-frequency hearing loss with absent otoacoustic emissions.

At 6 years of age, she has cerebellar signs, which include gait ataxia, peripheral ataxia with dysdiadochokinesia, dysmetria, and titubation. Her reflexes are exaggerated with ankle clonus but flexor plantar response. She has everted and plantar-flexed feet, but no pes cavus or scoliosis. There is no nystagmus with normal vision and extraocular movements.

#### Patient 1-4

This female is the youngest of three siblings (patients 1-2, 1-3). She first presented at 4 months of age with apparent GTCS occurring during sleep. Since then, she has received valproate, as did her sister, with complete seizure control. Electrolytes at presentation were normal, with borderline low potassium (3.8mmol/L) and magnesium (0.73mmol/L) levels. Hypomagnesaemia has been noted intermittently since 10 months of age.

An intention tremor was noted from a very early age. She has truncal ataxia but she seems to be less affected than her other two siblings. She has been walking independently since 3 years 6 months of age. Her gait is broad based, but she is the most confident of her siblings, and has shown gradual developmental progress. Her first meaningful words were at 1 year of age. She is speaking full sentences at present but with slurring of speech and an explosive tone. She attends a mainstream school.

Like her siblings, a hearing problem was not recognized, but audiological testing at the age 4 years revealed high-frequency hearing loss with absent otoacoustic emissions.

On examination at 4 years of age, she has a broad-based gait with tremors, head titubation, dysmetria, and dysdiadochokinesia. Her reflexes are normal, she has no ankle clonus, and she has a flexor plantar response. She has no nystagmus. Audiological testing revealed mild sensorineural impairment.

#### Patient 2

This male of Pakistani heritage, reviewed at 16 years, is the youngest of three siblings, the older two being fit and well. The family migrated to the UK when he was 9 years old and there are very limited medical records available from before this time. He was born in Pakistan at term after an unremarkable pregnancy with a birthweight of 3.1kg.

The family first lived in the United Arab Emirates, where he presented at 5 months of age with a seizure that occurred from sleep, involving stiffening of arms with facial twitching, and loss of consciousness lasting 5 minutes. Phenobarbital treatment was commenced for seizure control, and was replaced with valproate at 6 months. Subsequently, he developed further apparent GTCS at 9 months of age, lasting 20 minutes. However, he remained seizure free from 9 months to 12 years of age and was weaned from valproate at 4 years. At 12 years of age, he developed a tonic–clonic seizure (TCS) from sleep with left-sided predominance including left-sided facial twitching, lasting 20 minutes. Valproate was recommenced. He currently experiences two types of seizures; first, two or three daily episodes of behavioural arrest associated with lip smacking and loss of awareness, lasting up to 2 minutes and suggestive of focal seizures; second, TCS which occur once every 6 months with a tendency to be prolonged, and on two occasions resulted in intensive care admissions for status epilepticus. Both seizure types have continued despite treatment with phenytoin and valproate.

He was hospitalized at 2 years of age with a 4-day history of fever and vomiting. On admission, serum sodium and potassium levels were 121mmol/L and 1.1mmol/L respectively, leading to a diagnosis of a tubulopathy. He was prescribed potassium supplements, indometacin, and spironolactone.

Over time, an unstable broad-based gait with frequent falls has manifested. On examination he has an intention tremor, dysdiadochokinesia, and dysmetria. He has exaggerated lower limb reflexes, with an ankle clonus of 8 to 10 beats and extensor plantars. There is dystonic posturing of the upper limbs on walking.

His development has been delayed: he crawled at 1 year of age, walked independently at 2 years 6 months of age, and currently falls frequently while walking. He spoke his first meaningful words at 2 years of age, with current slow and slurred speech. He is in special education. EEG at 13 years while on valproate and phenytoin medication showed waves of low amplitude and low frequency. No epileptiform discharges were seen, but a mild excess of slow-wave activity was observed, intermixed in the posterior regions with 2 to 3Hz sharp-wave activity. Repeat EEG at 16 years was normal.

He was suspected of having a hearing problem at the age of 15 years. Subsequent audiological testing revealed sensorineural hearing loss.

#### Patient 3

This male is of Iranian heritage and his first reported possible seizure occurred during the neonatal period, with generalized stiffening for less than 20 seconds, with spontaneous resolution. At 2 months, further seizures were reported but no further details are available. Treatment with phenobarbital led to seizure control. Subsequently he continued to have a TCS once every 6 to 12 months, always during sleep, which responded to a weight-adjusted dose of phenobarbital. Phenobarbital treatment was maintained until 7 years of age, when it was replaced with lamotrigine with subsequent seizure control.

At 6 years of age, he presented with vomiting and lethargy associated with a viral illness and was noted to have a potassium level of 2.2mmol/L. Subsequent measurements and 24-hour urine sampling reportedly demonstrated renal potassium loss and a tubulopathy was suspected. The parents state that whenever he had a gastrointestinal illness and low potassium values, he had difficulties in moving. Magnesium losses were noted later.

He learned to walk by 7 years of age with a broad-based gait and ataxia and has not deteriorated. He received regular physiotherapy from 3 until 14 years of age, requiring splints, Pedro boots, insoles, and orthotics. A tremor was first noticed at 3 years 6 months of age, this being worse in the morning. He was noted to frequently spill from a cup, and is not able to use a fork at present. He can use zippers, but is unable to do up buttons. He uses a computer to aid his writing skills.

Speech was slurred and slow from an early age and is, at present, explosive. He spoke his first word at 3 years, and at 16 years has a total of 30 spoken words. He has received regular speech therapy throughout his school years.

His parents noted that he did not respond to noises from as early as 1 to 2 years of age, and a formal hearing test at 5 years revealed sensorineural deafness requiring hearing aids. Examination showed ataxia with past-pointing and dysdiadochokinesia; he has generalized increased tone with brisk deep tendon jerks and extensor plantar response.

#### Patient 4

This male presented aged 2 months with generalized stiffening of the whole body, occurring on awakening. He was adopted as an infant and family history and ethnicity are unclear, as are further details of his early seizure history. He was extensively investigated at 18 months of age and was treated with carbamazepine, which was later replaced with lamotrigine. He continues to have apparent GTCS requiring occasional hospitalization. At 5 years, biochemical investigations revealed a hypokalaemic, hypochloraemic metabolic alkalosis. He was noted to have developmental delay at 2 years 6 months of age, with evidence of ataxia. He achieved walking with the aid of a walking frame. Since the age of 11 years, he has had difficulties supporting his weight and has been using a wheelchair. He had speech delay; sensorineural deafness was detected at age 2 years 6 months necessitating a hearing aid. He is currently able to speak in short simple sentences. No formal speech assessments have been performed. He has been in special education throughout his school years and at 20 years of age lives in a residential college with the aim of acquiring independence skills.

At 20 years of age, he exhibits past-pointing and dysdiadochokinesia with a scanning dysarthric speech. His deep tendon reflexes are present, but not exaggerated. There is a suggestion of dystonic posturing of the fingers when stressed.

#### Patient 5-1

The older of two siblings of Afro-Caribbean origin, this female presented at 3 months of age with a reported GTCS. Treatment with valproate was initiated. Seizures were not considered problematic, and she remained seizure free from 4 to 8 years of age. On an attempted wean from valproate, she was troubled by frequent TCS as well as non-convulsive status epilepticus. Control was not regained on reintroduction of valproate, but she became, and remains, seizure free on lamotrigine.

She was not suspected of having a hearing problem, but when screened at the age of 15 years, after her younger brother (patient 5-2) was diagnosed with hearing loss, she was also found to have high-frequency hearing loss.

Electrolytes were first measured at the age of 18 years and revealed hypokalaemic alkalosis and hypomagnesaemia.

She was noted to have increased tone and unsteadiness from infancy. She did attain independent walking at 17 months; however, with the deterioration in seizure control at 8 years she lost mobility and this has not been regained. Her gait shows elements of ataxia; she has intention tremor, dysmetria, and dysdiadochokinesia. There is also apparent dystonia with bradykinesia. There is increased tone in the lower limbs, and no clear clonus, but there are extensor plantar responses. She has a scoliosis. She also has global cognitive difficulties, with the gap widening between her and her peers over the years (18y of age at last review); however, there has been no period of loss of cognitive skills.

#### Patient 5-2

This male is the younger brother of patient 5-1. He experienced onset of seizures at 6 months with a self-limiting GTCS. He subsequently had three afebrile seizures at 9 months. He then had no further seizures until 4 years of age, when he developed left focal seizures. These responded to lamotrigine after failed trials of valproate and levetiracetam.

Plasma electrolytes were first checked at initial presentation and included a normal plasma potassium value of 4.4mmol/L. At the age of 5 years, plasma potassium was borderline low at 3.8mmol/L. Magnesium was not tested on either occasion. At 10 years of age he has hypokalaemia (3.3mmol/L), but normal plasma magnesium (0.74mmol/L).

His hearing was first tested at the age of 4 years and revealed sensorineural deafness for which he was fitted with hearing aids.

He began to walk late: at 2 years of age. At the age of 10 years he has an ataxic gait but is able to run. He has intention tremor and dysmetria. His speech also has a cerebellar quality. Muscular tone is symmetrically increased but there is no clonus and flexor plantar response. He is cognitively behind his peers, but making progress.

### Epilepsy

All children in the above cases presented with seizures early in the first year of life. These were all described in the records as GTCS, although it could not be determined whether they were primarily or secondarily generalized. In six of the patients, epilepsy was initially controlled with antiepileptic drugs (AEDs) at presentation; the three others were still experiencing seizures at admission but they later achieved remission. In all but the youngest two patients (6y 4.8mo and 4y) focal seizures later re-emerged (with or without progression to bilaterally convulsive seizures), but were readily controlled with AEDs in four of the patients. One underwent video-EEG telemetry to document events from sleep at 16 years; all events were stereotyped but there was no consistent EEG change. Despite the unremarkable EEG findings, the consistent stereotyped nature led to a diagnosis of frontal lobe seizures, which did indeed respond to a single AED. Lamotrigine has been the effective medication when trialled in three children, whereas two others responded to valproate. Three children continue to have drug-resistant seizures of apparent focal onset. There was no apparent relationship between drug resistance and age.

### Neurological development and motor disorder

All children had evidence of early motor delay in their history. Two children (patient 1-2 and patient 4) have never achieved independent walking as a result of ataxia. Age at walking in others ranged from 17 months to 7 years. All have been unsteady to a variable degree with frequent falls from the onset of walking, suggesting ataxia from the outset, with only two patients (patient 5-1 and patient 5-2) reporting a history of steady deterioration. Patient 5-1 walked at 17 months, but this was lost after a prolonged seizure at 8 years. All children were reviewed at a single point in time rather than on serial examinations. However, even within the same family, there is a degree of phenotypic variability, with no obvious relationship between severity of motor impairment and age. In family 1, the three siblings (patient 1-2, patient 1-3, and patient 1-4) were reviewed at 9 years, 6 years, and 4 years. The middle child walked at 6 years and retained the skill, whereas the oldest child has never walked and the youngest achieved independent walking at 2 years.

All the patients who are ambulant walk with a broad-based ataxic gait. All show evidence of dysmetria, dysdiadochokinesia, and intention tremor. One has scoliosis. All have brisk deep tendon jerks, but all except two (patient 13 and patient 2) show no evidence of clonus. There is the suggestion that tone increases with age, with dystonic posturing more evident in the older children.

Detailed neuropsychological assessments have not been carried out, but all children have shown a degree of cognitive delay since the first year of life, with all demonstrating mild to moderate learning difficulties at clinical review. All have shown steady cognitive improvement, albeit slow when compared with their peers. Five have a statement of special educational needs.

### Investigations

#### Neuroimaging

**Figure 1:**
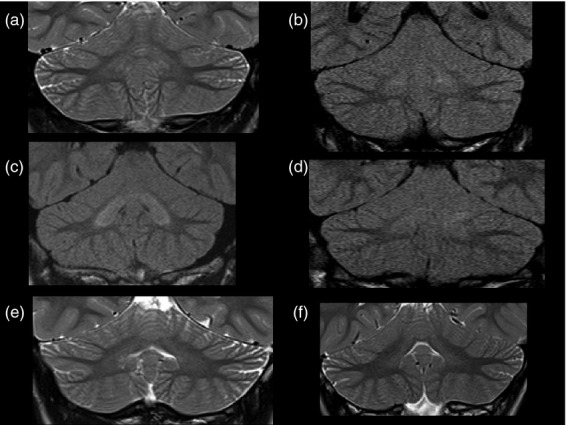
Patients with EAST syndrome show increased signal intensity of the cerebellar nuclei and cerebellar hypoplasia on brain magnetic resonance imaging. Coronal T2 or fluid-attenuated inversion recovery images show varying degrees of signal hyperintensity in the cerebellar deep nuclei. These ranged from subtle signal changes in comparison with the cerebellar cortex to more obvious changes within both the cerebellar deep nuclei and hilar white matter. (a) patient 1-1, (b) patient 2, (c) patient 4, (d) patient 3, (e) patient 5-1, and (f) patient 5-2.

**Figure 2:**
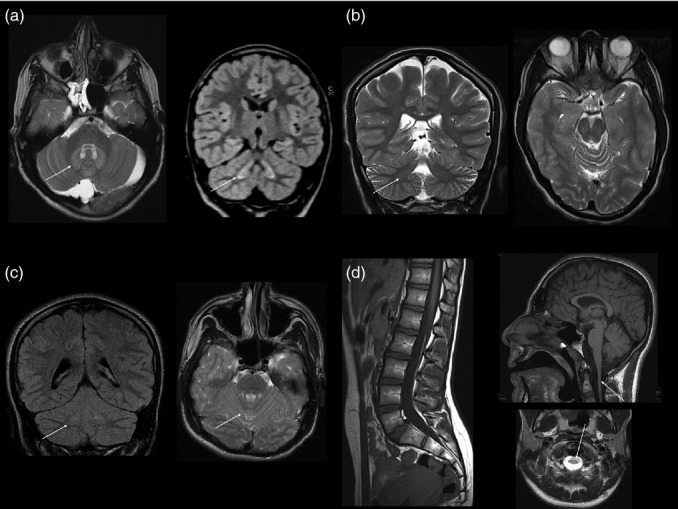
Other abnormalities on brain magnetic resonance imaging. (a) Axial T2-weighted and coronal volumetric fluid-attenuated inversion recovery images in patient 3 show bilateral symmetrical signal hyperintensity within the cerebellar deep nuclei and hilar white matter (arrow) and a retrocerebellar arachnoid cyst. This child also has a fatty filum terminale but normal position of the conus medullaris and no other spinal dysraphic features (not shown). (b) Coronal and axial T2-weighted images in patient 5-2 show prominence of the cerebellar hemisphere and vermian fissures. Note again the subtle symmetrical dentate nucleus changes (arrow). (c) Sagittal T1-weighted image (patient 1-1) shows normal position of the conus medullaris, a fatty filum terminale, and normal spinal cord volume. (d) Sagittal T1- and axial T2-weighted brain images (patient 2) show global lack of cerebral volume with slightly thin corpus callosum and brainstem and spinal cord hypoplasia.

Neuroimaging was available for review in six of the nine children; complete brain and spine MRI was conducted in three and brain MRI alone or with additional limited spine imaging was conducted in the other three. Two children underwent repeat brain MRI (8y and 10y after the initial scan), and these showed no evidence of progressive change. Age at imaging was ranged from 9 to 19 years. MRI in all six children showed symmetrical signal abnormality of varying severity in the cerebellar dentate nuclei and dentate hila (see Fig. 1). All six also showed evidence of cerebellar hypoplasia, of varying severity. The cerebellar hypoplasia was most severe in patient 5-1, affecting the cerebellar vermis and both cerebellar hemispheres, with the pons appearing normal. Four children had a thin corpus callosum, reflecting the lack of white matter bulk. The same four children had some degree of brainstem hypoplasia (see Fig. 2). Three children had a thin spinal cord (see Fig. 2). All three of the children with complete spinal imaging also had a fatty filum terminale with normally positioned conus medullaris (see Fig. 2).

##### Quantitative image analysis

**Figure 3:**
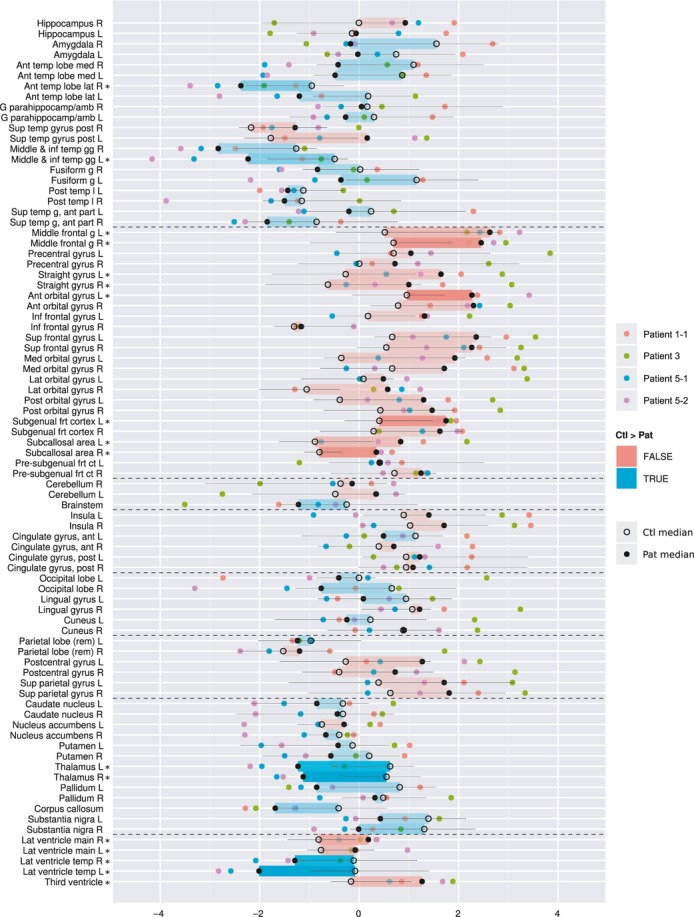
Comparative size of individual regions in patients (Pat) and matched comparison children (Ctl), represented as z-scores, using the IXI cohort as a normative reference. Coloured discs, individual patients; black discs, patient median; rings, comparison median; black lines, interquartile range of comparison group. Coloured rectangles indicate whether the patient median z-score is larger (red) or smaller (blue) than the comparison median z-score. Regions where the difference is significant (as determined by a Wilcoxon unpaired ranked-sum test, alpha=0.05, no correction for multiple comparisons) are marked with an asterisk (*). The colour intensity of the rectangles also represents significance. Regions are grouped anatomically, with groups separated by dotted lines. From top to bottom: temporal lobe, frontal lobe, cerebellum and brainstem, insula and cingulate gyri, occipital lobe, parietal lobe, central structures, ventricles. R, right; L, left; ant, anterior; temp, temporal; med, medial; lat, lateral; sup, superior; post, posterior; frt, frontal; inf, inferior; ct, cortex; g, gyrus; gg, gyri; l, lobe; rem, remainder; G parahippocamp/amb, gyrus parahippocampalis et ambiens.

Morphometric analysis of the brain reveals a systematic influence that EAST pathology appears to have on the entire brain's anatomical configuration (Fig. 3). Consistent with the radiological interpretation of the MRI, region volumes in the brainstem and corpus callosum were reduced. Variations in ventricular size were evident, with large lateral ventricles and small temporal horns. Reductions in thalamic volume were also substantial.

#### Audiology

A detailed hearing assessment was performed in eight children, using tympanometry, pure-tone audiometry, and otoacoustic emissions. In only six patients was hearing loss clinically suspected before testing; audiometry revealed high-frequency hearing loss in all eight patients assessed; whereas tympanometry was normal in all. Otoacoustic emissions could be assessed in five children and were absent in all.

#### Other investigations

These included EEG (*n*=5), nerve conduction studies (*n*=5), and metabolic evaluation. EEG was abnormal on only one occasion in one patient, showing spikes and sharp waves with slow activity over one hemisphere; subsequent EEG was normal. The remaining investigations performed revealed no abnormality. Three children underwent muscle biopsy and one a skin biopsy, all of which were also reported histologically as normal. These studies were undertaken before the diagnosis of EAST syndrome was made.

## Discussion

A clinical syndrome characterized by epilepsy, ataxia, sensorineural deafness, and tubulopathy, EAST syndrome, has recently been linked to recessive mutations in the *KCNJ10* gene.^9^ Here we further delineate the neurological findings in nine children (including two sets of siblings). Key features include early-onset epilepsy, unrelated to the electrolyte imbalance, but readily controlled by antiepileptic medication. Although documented by description as generalized seizures, it remains unclear whether these were indeed secondarily generalized seizures with focal onset as might be expected in this age group.^21^ In many patients, the epilepsy is later characterized by more apparent focal seizures. Motor delay is apparent from the first year of life, with evident ataxia at the time of independent walking, if achieved. In most patients, the ataxia appears to be persistent and non-progressive, although in one child definitive deterioration of motor function was seen, possibly related to the occurrence of a prolonged seizure. There is the impression of increasing tone with age, with dystonic posturing seen in older children. Thus, EAST syndrome appears to have both static and progressive features, in contrast to the episodic or self-limiting disease seen in other potassium channelopathies with neurological features.^22^

Ion channel mutations are being increasingly associated with specific epilepsy syndromes. These syndromes may include single or multiple seizure types, and may have a benign or a more complex course.^23^ Notably, mutations in the potassium channel gene *KCNQ2* have been linked to benign familial neonatal convulsions, a syndrome in which seizures are seen in the neonatal period only and are self-limiting, with normal subsequent neurological development.^24^ The patients described here, all with mutations in the gene encoding the inwardly rectifying potassium channel, *KCNJ10*, presented in the first year of life with isolated apparent GTCS, although in this age group focality of onset may be difficult to determine.^21^,^25^ These initial seizures, on the whole, responded well to treatment with antiepileptic drugs; however, the possibility of a biphasic presentation, initially self-limiting but with re-emergence of seizures at a later age, needs to be considered. Later presentation appears to involve more definitive focal seizures (of differing semiology between individuals) and, again, is relatively easily controlled with antiepileptic medication in most patients.

Inwardly rectifying potassium channels such as KCNJ10 are thought to be involved in the spatial buffering of potassium by mediating potassium movement from areas of high extracellular concentration, thus lowering neuronal excitability.^26^ Genetic variations in KCNJ10 have been previously implicated in seizure susceptibility in idiopathic generalized epilepsy syndromes.^27^,^28^ Notably, lamotrigine, which is known to suppress repetitive firing through inactivation of voltage-gated sodium channels once cells are depolarized,^29^ was effective in three individuals in whom it was trialled, thereby linking the clinical effect to a possible mode of action.

The motor disorder seen in our patients is difficult to characterize. Ataxia was seen in all, manifesting from the time of walking and appearing non-progressive in most. Manifestations of poor pelvic control were demonstrated in those who were non-ambulant, giving the impression of a peripheral muscular disorder, but nerve conduction studies as well as muscle biopsy were normal where they were performed. Further, increasing tone was seen with age, as dystonic posturing was seen in the older children. Cognitive abilities appeared impaired in some, but did not deteriorate. Early and severe ataxia can affect verbal and written communication, and therefore may have confounded the assessment. Thus, the term intellectual disability* is not appropriate for all patients with EAST syndrome.^30^

Visual MRI analysis, when available, showed consistent abnormalities with respect to cerebellar volume (suggestive of hypoplasia) and signal change in the dentate nuclei. Such changes, therefore, could be put forward as part of the clinical diagnostic criteria. Further, they provide a structural correlate to the ataxia. In addition, brainstem hypoplasia and a thin corpus callosum were seen in four of the six patients and spinal cord abnormalities were seen in three. The identification of these abnormalities is in contrast to our initial report, in which MRI in two patients was considered normal.^9^ This demonstrates the difficulties of assigning the judgement of normal or abnormal to subtle changes. If seen in an isolated patient, such changes may represent a variant of normal; however, if the changes are present in several or all patients with the same disorder, they are likely to represent a feature of the disease. Indeed, the unbiased and detailed morphometric analysis confirmed several of these changes: the brainstem and the corpus callosum are significantly smaller in patients than in comparison children. In addition, it revealed systematically smaller volumes in temporal cortical regions, as well as larger volumes in the frontal lobe. These findings have to be interpreted with care, because of the small number of participants, and because we did not correct for multiple comparisons after applying the Wilcoxon ranked-sum test to each individual region. Despite these caveats, the impression of systematic change from the visualization in Figure 3 is distinct. It is not clear how dysfunction of KCNJ10 causes these morphological abnormalities, but the channel has been implicated in myelination in mice. KCNJ10 knockout mice present with profound ataxia and late stimulus-sensitive seizures, with early mortality.^13^,^31^

The volumetric analysis is complementary to visual analysis and no replacement for expert neuroradiological assessment: in the case of clear volumetric abnormalities, these will be detected by both analyses (e.g. patient 3; Figs 1d and 3 [individual volumes for this child]). Pure FLAIR signal changes without volumetric repercussion, or widened cerebellar sulci less than a voxel wide (e.g. patient 5-2; Fig. 1f) will be easily detectable by visual analysis but invisible to MAPER. Subtle changes in absolute volume, however, are very hard to detect visually. The presence of such systematic volumetric change in various brain areas is strongly suggested from the visualization of the MAPER results in Figure 3, demonstrating the power of this approach.

## Conclusion

Our study raises several important points concerning the clinical features of EAST syndrome. First, we found that infantile onset of seizures was a consistent presentation in all the patients studied. This was followed by the later re-emergence of more definitive focal seizures. In general, however, the prognosis for seizure control appears good. Second, high-resolution MRI revealed consistent cerebellar changes in all patients examined, providing a further diagnostic tool, as well as a likely morphological correlate to the ataxia. Third, automatic brain image analysis also revealed a characteristic pattern of morphological change. Fourth, we identified no clear evidence for a progressive course of the disease, an important point invariably raised by affected families, who want to know what to expect for the future. However, longitudinal studies of individual EAST patients are required to clarify this point.

## Footnotes

* North American usage for mental retardation

## Abbreviations

AED: Antiepileptic drugs
EAST: Epilepsy, ataxia, sensorineural deafness, and tubulopathy
GTCS: Generalized tonic–clonic seizure
MAPER: Multi-atlas propagation with enhanced registration
TCS: Tonic–clonic seizure

## SUPPORTING INFORMATION

**Video S1:** Video of patient EAST 5-2 at age 10 years demonstrating ataxic gait, dystonic posturing of the upper limbs on stressed walking, and intention tremor.

**Figure S1:** Brain regions are correctly identified by automatic segmentation. Axial, coronal, and sagittal sections of T1-weighted image of patient 5-1 show examples of anatomical regions as identified by the automatic segmentation algorithm, highlighted by coloured contours.
