# Supplementary figures and images for: Neurological features of epilepsy, ataxia, sensorineural deafness, tubulopathy syndrome
Source: Dev Med Child Neurol. 2013 Mar 14;55(9):846–56. doi: 10.1111/dmcn.12171 (PMC4298033; doi:10.1111/dmcn.12171)

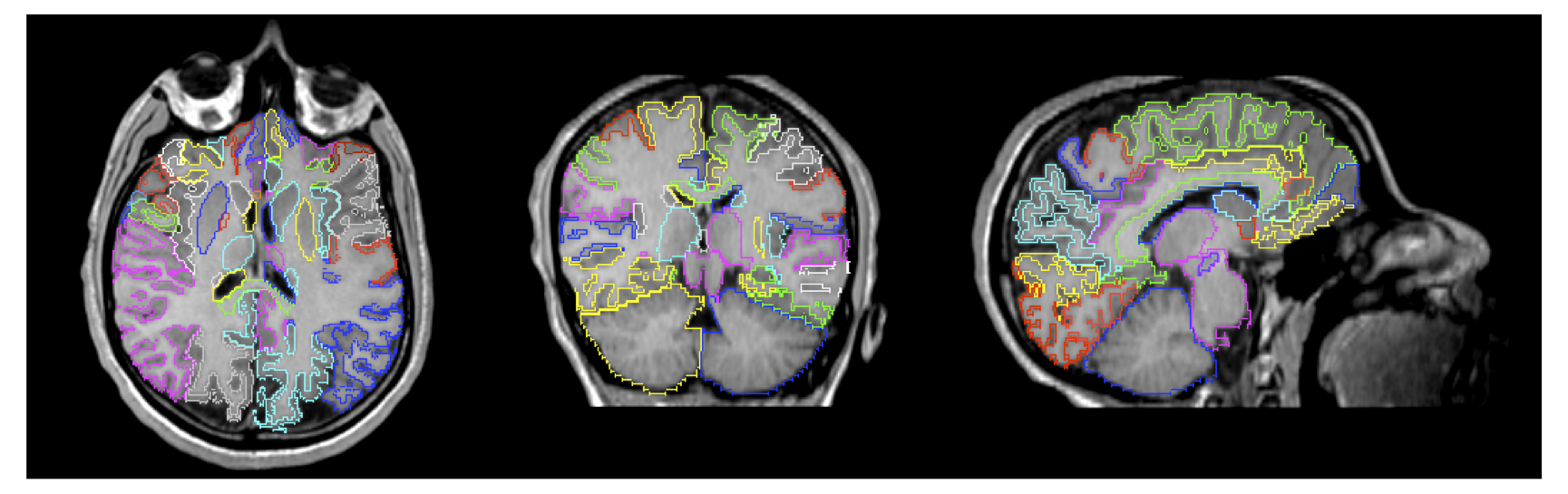

Supplement: Supplementary file 2 — Figure S1: Brain regions are correctly identified by automatic segmentation. Axial, coronal, and sagittal sections of T1-weighted image of patient 5-1 show examples of anatomical regions as identified by the automatic segmentation algorithm, highlighted by coloured contours. [file dmcn0055-0846-sd2.tif]
